# *MYCN* amplification confers enhanced folate dependence and methotrexate sensitivity in neuroblastoma

**DOI:** 10.18632/oncotarget.3732

**Published:** 2015-03-30

**Authors:** Diana T. Lau, Claudia L. Flemming, Samuele Gherardi, Giovanni Perini, André Oberthuer, Matthias Fischer, Dilafruz Juraeva, Benedikt Brors, Chengyuan Xue, Murray D. Norris, Glenn M. Marshall, Michelle Haber, Jamie I. Fletcher, Lesley J. Ashton

**Affiliations:** ^1^ Children's Cancer Institute Australia, Lowy Cancer Research Centre, Randwick, NSW, Australia; ^2^ Department of Biology, University of Bologna, Bologna, Italy; ^3^ Children's Hospital, Department of Pediatric Oncology and Hematology, University of Cologne and Centre for Molecular Medicine Cologne, Cologne, Germany; ^4^ Division of Theoretical Bioinformatics, German Cancer Research Center, Heidelberg, Germany; ^5^ Kids Cancer Centre, Sydney Children's Hospital, Randwick, NSW, Australia; ^6^ Faculty of Medicine, School of Women's and Children's Health, University of New South Wales, Sydney, NSW, Australia and Research Portfolio, University of Sydney, Sydney, NSW, Australia

**Keywords:** MYCN, MYC, SLC19A1, methotrexate, neuroblastoma

## Abstract

*MYCN* amplification occurs in 20% of neuroblastomas and is strongly related to poor clinical outcome. We have identified folate-mediated one-carbon metabolism as highly upregulated in neuroblastoma tumors with *MYCN* amplification and have validated this finding experimentally by showing that *MYCN* amplified neuroblastoma cell lines have a higher requirement for folate and are significantly more sensitive to the antifolate methotrexate than cell lines without *MYCN* amplification. We have demonstrated that methotrexate uptake in neuroblastoma cells is mediated principally by the reduced folate carrier (RFC; *SLC19A1*), that *SLC19A1* and *MYCN* expression are highly correlated in both patient tumors and cell lines, and that *SLC19A1* is a direct transcriptional target of N-Myc. Finally, we assessed the relationship between *SLC19A1* expression and patient survival in two independent primary tumor cohorts and found that *SLC19A1* expression was associated with increased risk of relapse or death, and that *SLC19A1* expression retained prognostic significance independent of age, disease stage and *MYCN* amplification. This study adds upregulation of folate-mediated one-carbon metabolism to the known consequences of *MYCN* amplification, and suggests that this pathway might be targeted in poor outcome tumors with *MYCN* amplification and high *SLC19A1* expression.

## INTRODUCTION

Folate is essential for DNA synthesis and cell growth in rapidly dividing cells. In cancer, folate uptake and metabolism are frequently up-regulated to meet the elevated need for nucleotides. Inhibition of folate metabolism is the basis for several widely used cancer drugs, including methotrexate [1-3], which exerts its toxicity by inhibiting dihydrofolate reductase (DHFR) thereby disrupting purine and thymidylate biosynthesis, inhibiting DNA replication and promoting cell death. This folate antagonist has been used as a chemotherapeutic agent to treat a variety of hematological malignancies and solid tumors including acute lymphoblastic leukemia, non-Hodgkin's lymphoma, osteosarcoma, head and neck cancer, choriocarcinoma, small cell lung cancer, and breast cancer [4].

In the 1970s, clinical trials of methotrexate treatment in children with neuroblastoma showed high levels of toxicity and low response rates [5, 6]. No further clinical trials examining the efficacy of methotrexate treatment have been undertaken in neuroblastoma and methotrexate is currently not considered a treatment option for children with this disease. However, these early clinical trials included only small numbers of patients and were conducted in an era when the prognostic value of *MYCN* testing was yet to be established. Amplification of the *MYCN* oncogene is now known to occur in approximately 20% of primary neuroblastomas and is consistently associated with poorer clinical outcome [7, 8]. Similar to other members of the Myc family, N-Myc is an oncogenic transcription factor that dimerizes with partner proteins and binds at E-box sequences (6 nucleotide binding motif) within the promoter of target genes [9]. N-Myc expression has been shown to correlate with growth potential of neuroblastoma cells [10, 11], and may promote an aggressive tumor phenotype through regulation of genes particular to the cell cycle, DNA damage response, differentiation and apoptosis [12]. Relatively little is known about the relationship between folate metabolism and neuroblastoma biology, however folate-mediated one-carbon metabolism has previously been identified as correlating with neuroblastoma aggressiveness in bioinformatics analyses [13], suggesting that inhibition of folate metabolism may be beneficial in high-risk disease.

The *SLC19A1* gene encodes the reduced folate carrier (RFC), a high capacity reduced folate transporter that is the most efficient of the cellular folate uptake mechanisms [3]. RFC is also the principle uptake mechanism for the antifolate methotrexate [3] and decreased RFC activity has been shown to be one of the most common mechanisms of methotrexate resistance in osteosarcoma [14-16]. RFC is ubiquitously expressed in rapidly dividing fetal and tumor cells, reflecting its essential role in supplying folate cofactors for DNA synthesis and cell proliferation. As a previous large-scale screen suggested that *SLC19A1* can be regulated by Myc [17], and N-Myc and Myc are largely functionally equivalent [18], we hypothesized that *SLC19A1* would also be regulated by N-Myc and would be expressed at elevated levels in *MYCN*-amplified tumors, allowing effective methotrexate uptake.

This study investigated the reliance of *MYCN*-amplified neuroblastoma cell lines on folates, their sensitivity to the antifolate methotrexate and the contribution of RFC to methotrexate uptake. The relationship between *SLC19A1* and *MYCN* expression was examined in tumors from two independent patient cohorts, in a panel of 13 neuroblastoma cell lines and in cell lines with inducible and silenced *MYCN* expression. The regulation of *SLC19A1* by N-Myc was examined using quantitative chromatin immunoprecipitation (ChIP) and luciferase reporter assays. The association between *SLC19A1* gene expression levels and risk of relapse or death was also examined in patient samples.

## RESULTS

### *MYCN*-amplified neuroblastoma cells have higher folate requirements than non-amplified cells

**Figure 1:**
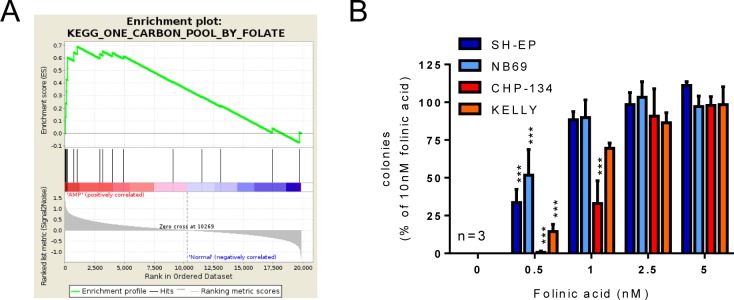
*MYCN*-amplified cell lines have a higher requirement for folate than those without amplification (**A**) GSEA enrichment plot for the KEGG “One carbon by folate” pathway, showing correlation with *MYCN* amplification. Genes from this pathway are over-represented at the top of the whole gene list when ranked based on their relationship to *MYCN* amplification status. (**B**) Colony formation for *MYCN* single copy (SH-EP, NB69) and *MYCN*-amplified (CHP-134, Kelly) cell lines in a range of folinic acid concentrations. Colony numbers are normalised to 10 nM folinic acid at 100%, and comparisons made to the 5 nM folinic acid values. Data show means and SD derived from three independent experiments.

To gain insight into the biological pathways upregulated in *MYCN*-amplified neuroblastoma, Gene Set Enrichment Analysis [19] was performed on pre-existing microarray data from a cohort of tumors from 650 neuroblastoma patients [20]. Using a false discovery rate (FDR) of 0.25 as a cut-off, 20 pathways were positively correlated with *MYCN* amplification (Supplementary Table S3). The most highly enriched gene sets were those for the Kyoto Encyclopedia of Genes and Genomes (KEGG) pathways “Ribosome” (ribosome components), “DNA replication” (DNA replication machinery), “Base excision repair” (base excision repair machinery), “One carbon pool by folate” (folate-mediated one-carbon metabolism) and “RNA polymerase” (RNA polymerase subunits), each of which had a FDR less than 0.05 and an Enrichment Score (ES) greater than 0.65. Folate-mediated one-carbon metabolism (Figure 1A) was of particular interest having previously been identified as correlating with neuroblastoma aggressiveness in bioinformatics analyses [13], and being targetable by existing agents such as methotrexate.

To test whether upregulation of the folate-mediated one-carbon metabolism pathway corresponds to an increased reliance on folate in *MYCN*-amplified cells, we assessed the colony forming ability of two *MYCN*-amplified cell lines (CHP-134 and Kelly) and two non-amplified lines (SH-EP and NB69) in folate-free medium supplemented with increasing concentrations of folinic acid. As expected, none of the cell lines formed colonies in the complete absence of folates (Figure 1B). In the presence of 0.5 nM folinic acid, the *MYCN*-amplified lines formed few colonies (1% of the 10 nM value for CHP-134 and 14% for Kelly; *P* < 0.001 for each in comparison to 5 nM folinic acid) while the non-amplified lines exhibited partial rescue of colony formation (34%, *P* < 0.001 for SH-EP and 52%, *P* < 0.001 for NB69). In the presence of 1 nM folinic acid the *MYCN*-amplified lines exhibited partial rescue (33% of the 10 nM value, *P* < 0.001 for CHP-134 and 70%, *P* = 0.256 for Kelly) while the non-amplified lines exhibited complete rescue (90% of the 10 nM value, *P* = 0.453 and *P* = 0.410 for SH-EP and NB69 respectively). *MYCN*-amplified neuroblastoma cells therefore appear to have a greater requirement for folates than their non-amplified counterparts.

### *MYCN*-amplified neuroblastoma cells have enhanced methotrexate sensitivity, with uptake mediated by RFC

**Figure 2:**
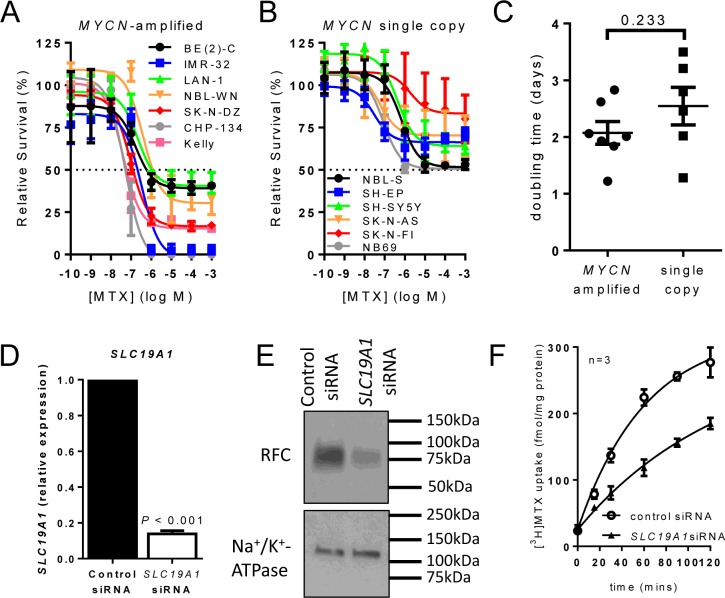
*MYCN*-amplified cell lines are more sensitive to methotrexate than non-amplified lines, with Reduced Folate Carrier (SLC19A1) a major contributor to methotrexate uptake (**A**, **B**) Methotrexate dose-response curves at 72 h for a panel of 7 *MYCN*-amplified (A) and 6 *MYCN* single copy (B) cell lines showing the means and SD derived from three independent experiments. (**C**) Cell doubling times for *MYCN*-amplified and *MYCN* single copy cell lines do not differ significantly (*P* = 0.233, unpaired t-test). (**D**) Transfection of *MYCN*-amplified BE(2)-C cells with *SLC19A1* siRNA reduced *SLC19A1* expression 87% (7-fold) compared to control siRNA (paired *t*-test; *P* < 0.001). (**E**) Western blot of RFC protein levels in control and *SLC19A1* siRNA treated BE(2)-C cells. Representative results from triplicate experiments are shown, and full-length Western blots are shown in Supplementary Figure S1. (**F**) Knockdown of *SLC19A1* in BE(2)-C cells by siRNA halved the rate constant for [^3^H]methotrexate uptake from 0.016 min^−1^ to 0.008 min^−1^ (extra sum-of-square F test; *P* = 0.005). Data show means and SD derived from three independent experiments.

Folate-mediated one-carbon metabolism is targetable by the antifolate methotrexate. We therefore asked whether *MYCN*-amplified cells are more sensitive to methotrexate than those without *MYCN* amplification by generating dose-response data for a panel of 13 neuroblastoma cell lines, 7 of which were *MYCN*-amplified and 6 single copy. The IC_50_ for methotrexate in *MYCN*-amplified cell lines ranged from 0.05 to 1.18 μM (Figure 2A, Supplementary Table S4). In contrast, the methotrexate IC_50_ was not reached for any of the 6 single copy cell lines (Figure 2B, Supplementary Table S4) despite testing up to 1 mM methotrexate. To exclude the possibility that this striking difference was simply a consequence of more rapid proliferation in *MYCN*-amplified cell lines, we determined the doubling times of each cell line. The mean doubling times of *MYCN*-amplified and non-amplified lines were not significantly different across the cell line panel (Figure 2C, *P* = 0.233, Supplementary Table S4).

Since methotrexate uptake is mediated principally by the reduced folate carrier (RFC; *SLC19A1*) [3], we investigated the relevance of RFC expression for methotrexate uptake in neuroblastoma. Following down-regulation of *SLC19A1* in the *MYCN*-amplified cell line BE(2)-C using siRNA, *SLC19A1* mRNA expression was reduced 7-fold (paired *t*-test; *P* < 0.001; Figure 2D). At protein level, RFC expression was substantially reduced (Figure 2E, full-length Western blots presented in Supplementary Figure S1). As a result of RFC depletion, the rate constant for [^3^H]methotrexate uptake was reduced from 0.016 min-1 to 0.008 min-1 (Figure 2F, *P* = 0.005) indicating that RFC is a major contributor to methotrexate uptake in these cells.

### *SLC19A1* and *MYCN* expression are closely correlated in neuroblastoma tumors and cell lines

We subsequently investigated the expression of *SLC19A1* in primary neuroblastoma tumors using a 42 sample discovery cohort and a 650 sample validation cohort. As seen in Table 1, high levels of *SLC19A1* mRNA expression were significantly associated with *MYCN* amplification in both cohorts (Fisher's exact, *P* = 0.015 and *P* < 0.001 for the 42 and 650 tumor cohorts, respectively). In contrast, *SLC19A1* mRNA expression was not associated with sex, age or International Neuroblastoma Staging System (INSS) stage at diagnosis in the 42 tumor discovery cohort, but was associated with age and stage at diagnosis in the 650 sample validation cohort (*P* < 0.001). High *SLC19A1* expression therefore appears to be a common feature of *MYCN* amplified tumors.

**Table 1:** *SLC19A1* mRNA expression and clinical characteristics for the 42 sample discovery and 650 sample validation cohorts

	Discovery cohort (n=42) *SLCI9A1* mRNA^a^			Validation cohort (n=650) *SLCI9A1* mRNA^a^		
	High (%)	Low (%)	*P*^b^	High (%)	Low (%)	*P*^b^
**INSS Stage**						
1,2,3,4S	5 (33)	10 (67)	0.197	134 (39)	206 (61)	**<0.001**
4	16 (59)	11 (41)		188 (62)	116 (38)	
**Age at diagnosis**						
≤18 months	14 (42)	10 (58)	0.350	184 (44)	230 (56)	**<0.001**
>18 months	7 (61)	11 (39)		141 (60)	94 (40)	
**Sex**						
Male	12 (50)	12 (50)	1.000	165 (48)	179 (52)	0.443
Female	9 (50)	9 (50)		131 (49)	137 (51)	
***MYCN***						
Single copy	11 (37)	19 (63)	**0.015**	234 (43)	316 (54)	**<0.001**
Amplified	10 (83)	2 (17)		91 (93)	7 (7)	

a SLC19A1 mRNA levels were stratified into two groups around the median and designated as “high” or “low”.

b Calculated by Fisher's exact test.

**Figure 3:**
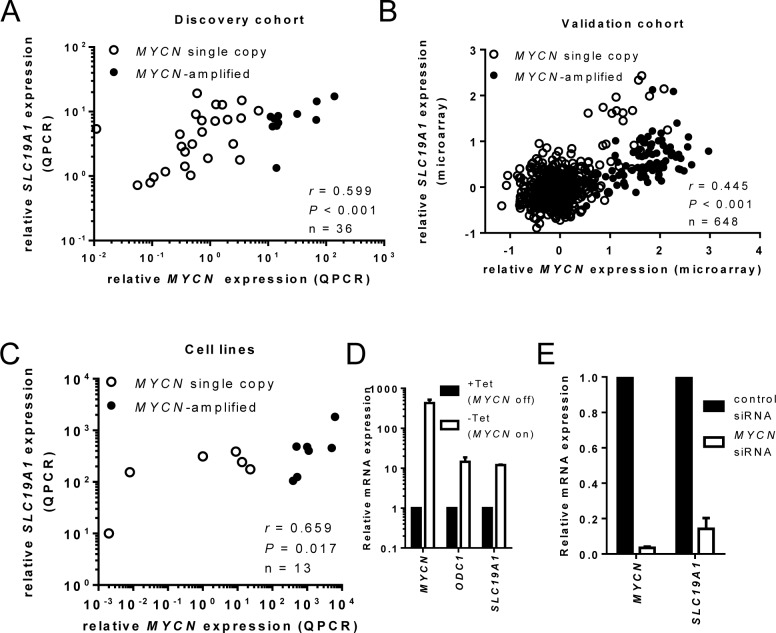
*MYCN* and *SLC19A1* expression are positively correlated in neuroblastoma tumors and cell lines *MYCN* and *SLC19A1* mRNA expression were positively correlated in (**A**) a panel of 37 neuroblastoma patients, (**B**) an independent cohort of 650 neuroblastoma patients and (**C**) 13 neuroblastoma cell lines. Log-transformed expression levels relative to calibrator are shown in A and C; log-transformed, zero-centred expression levels obtained from microarray dataset are shown in B. (**D**) *MYCN*, *SLC19A1* and *ODC1* expression in SH-EP Tet21N cells with tetracycline treatment (*MYCN* OFF), or without tetracycline (*MYCN* ON). *MYCN* induction (445-fold; paired *t*-test; *p* < 0.001) increased *SLC19A1* 12-fold (paired *t*-test; *P* < 0.001) and *ODC1* 14-fold (paired *t*-test; *P* < 0.001). (**E**) *MYCN* and *SLC19A1* expression in BE(2)-C cells transfected with control siRNA or *MYCN* siRNA. Silencing of *MYCN* (96.6% or 29-fold reduction in *MYCN* expression; paired *t*-test; *P* < 0.001) decreased *SLC19A1* expression 85.5% or 7-fold (paired *t*-test; *P* = 0.002). Bars represent the mean and SD from 3 independent experiments.

Next, we correlated expression of *MYCN* and *SLC19A1* following determination of *MYCN* mRNA levels in 36 of the 42 discovery cohort samples (insufficient cDNA for 6 samples). *MYCN* and *SLC19A1* expression showed a strong positive association (Figure 3A, Spearman's ρ = 0.599, *P* < 0.001). Similarly, a strong positive association was observed in the 650 sample validation cohort (Figure 3B, Spearman's ρ = 0.445, *P* < 0.001), and in the panel of 13 neuroblastoma cell lines (Figure 3C, Spearman's ρ = 0.659, *P* = 0.017).

The relationship between *MYCN* and *SLC19A1* expression was further investigated in *MYCN*-inducible Tet21N cells. Following removal of tetracycline, *MYCN* levels were induced 445-fold (Figure 3D, paired *t*-test, *P* < 0.001), accompanied by a 12-fold increase in *SLC19A1* expression (paired *t*-test, *P* < 0.001). The increase in *SLC19A1* was comparable to the 14-fold increase observed for *ODC1*, a well-established Myc family target gene [21] (paired *t*-test, *P* < 0.001). Conversely, down-regulation of *MYCN* in *MYCN*-amplified BE(2)-C cells using siRNA resulted in a 96.6% reduction in *MYCN* mRNA (Figure 3E, paired *t*-test, *P* < 0.001) accompanied by an 85.8% reduction in *SLC19A1* expression (paired *t*-test, *P* = 0.002).

### N-Myc regulates *SLC19A1* expression

**Figure 4:**
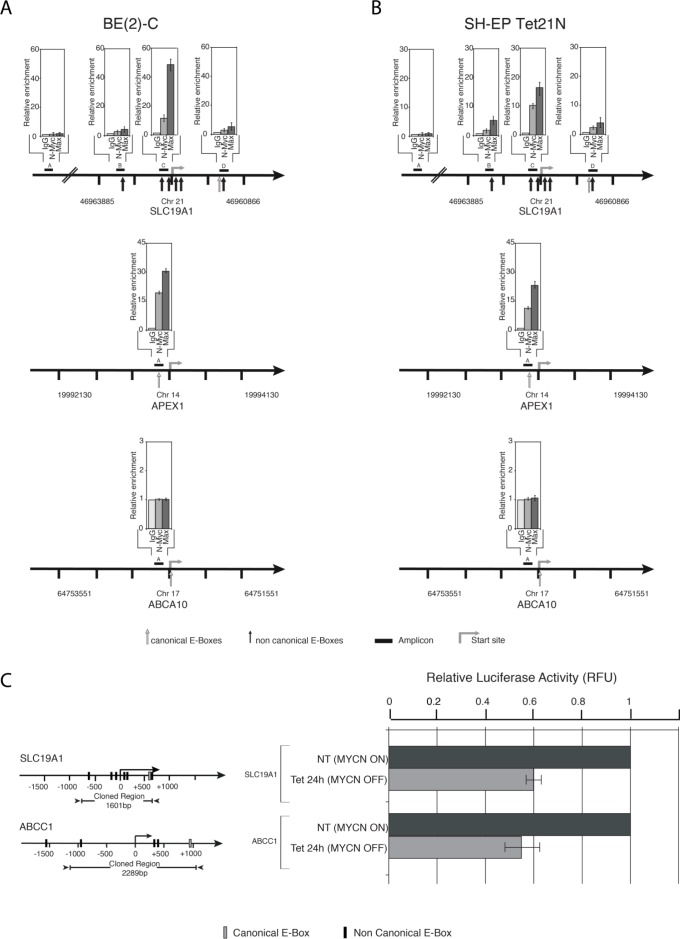
N-Myc is a direct transcriptional regulator of *SLC19A1* Quantitative ChIP was applied to (**A**) BE(2)-C cells and (**B**) SH-EP Tet21N cells following *MYCN* induction. Fold enrichment is relative to the pre-immune serum and results represent the mean and SD from three independent ChIP experiments in which each region was measured for expression using QPCR in duplicate. Both positive (*APEX1*) and negative (*ABCA10*) controls are shown. (**C**) N-Myc directly activates *SLC19A1* transcription. Luciferase activity was determined following transient transfection of *SLC19A1* reporter construct into SH-EP Tet21N cells in the absence (*MYCN* ON) and presence (*MYCN* OFF) of tetracycline for 12 hours (unpaired *t*-test, *P* = 0.002). *ABCC1* is included as a positive control.

To determine whether N-Myc and its binding partner Max associate with E-box sites in the promoter region of the *SLC19A1* gene in neuroblastoma cell lines, we employed chromatin immunoprecipitation (ChIP) assays. In both BE(2)-C and SH-EP Tet21N cells, N-Myc and Max bind to a region adjacent to the putative transcriptional start site which contains four non-canonical E-box sequences (Figure 4A–4B, amplicon C). Very weak enrichment was observed at the other two E-box-containing amplicons (amplicons B and D) and no enrichment was observed at the control amplicon (amplicon A) which does not contain an E-box. The fold enrichment for *SLC19A1* at amplicon C was comparable to that of the positive control *APEX1* in both BE(2)-C and *MYCN*-induced SH-EP Tet21N cells and no signal was detected for the negative control *ABCA10*.

The *SLC19A1* gene promoter was then cloned upstream of the luciferase reporter gene to determine whether N-Myc regulates *SLC19A1* transcription. Repression of N-Myc expression in SH-EP Tet21N cells reduced luciferase activity of the *SLC19A1* promoter/luciferase reporter construct with repression comparable to that observed for the established N-Myc target gene *ABCC1* [22] (Figure 4C). Collectively, these results indicate that *SLC19A1* is a direct transcriptional target of N-Myc in neuroblastoma cells.

### High *SLC19A1* expression is associated with an increased risk of relapse or death

**Figure 5:**
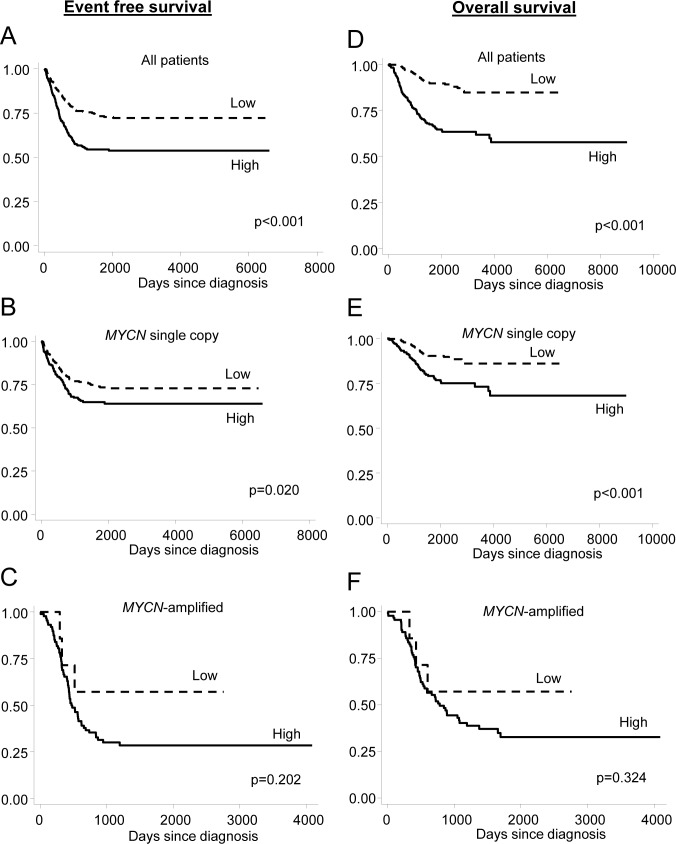
High *SLC19A1* expression is associated with an increased risk of relapse or death in neuroblastoma Kaplan-Meier curves showing the probability of EFS and OS for the entire 650 patient cohort (**A**, **D**), patients with tumors lacking *MYCN* amplification (**B**, **E**), and patients with *MYCN* amplification (**C**, **F**). Tumors were dichotomized into “high” and “low” *SLC19A1* expression groups around the median. Survival curves were analyzed by log-rank test.

Finally, we investigated the association between *SLC19A1* expression, established prognostic factors, and the risk of relapse and/or death in the cohort of 650 neuroblastoma patients. As expected, an increased risk of relapse and/or death was associated with *MYCN* amplification (*P* < 0.001), INSS Stage 4 (*P* < 0.001) and age >18 months at diagnosis (*P* < 0.001) (Table 2). When levels of *SLC19A1* mRNA expression were dichotomized around the median, higher levels of *SLC19A1* expression were strongly associated with reduced event-free survival (EFS) (HR = 1.98, 95%CI: 1.51–2.61; *P* < 0.001) with a 5-year survival rate of 72% ± 3% and 54% ± 3% in patients with low or high expression of *SLC19A1*, respectively. Similarly, high expression of *SLC19A1* was associated with poorer overall survival (OS) (HR = 3.66; 95%CI: 2.50–5.35; *P* < 0.001) with a 5-year OS rate of 85% ± 3% and 58% ± 4% in patients with low or high *SLC19A1* expression, respectively. Both EFS and OS were significantly lower in patients with higher *SLC19A1* expression in the entire cohort (Figure 5A, *P* < 0.001 and Figure 5D, *P* < 0.001) and in the subset of patients without *MYCN* amplification (Figure 5B, *P* = 0.020 and Figure 5E, *P* < 0.001). *SLC19A1* expression was not significantly associated with EFS or OS in patients with *MYCN*-amplified tumors (Figure 5C, *P* = 0.202 and Figure 5F, *P* = 0.324), most likely as the number of patients in the “low” category was very small. In multivariate analysis conducted on the entire cohort, high expression of *SLC19A1* was found to be independently associated with reduced EFS or OS after adjusting for *MYCN* amplification status, INSS Stage and age at diagnosis (EFS: HR = 1.36; 95%CI: 0.99–1.85; *P* = 0.054; OS: HR = 1.84; 95%CI: 1.19–2.83; *P* = 0.006; Table 2). High *SLC19A1* expression therefore appears to be a common feature of poor-outcome neuroblastoma, particularly in tumors with *MYCN* amplification.

**Table 2:** Univariate and multivariate analysis of prognostic factors and *SLC19A1* gene expression for event-free survival and overall survival (validation cohort)

		Event-free survival			Overall survival		
	Factor	Hazard ratio	95% CI	*P*	Hazard ratio	95% CI	*P*
**univariate**	Stage	4.0	3.0-5.5	<0.001	12.2	6.9-20.9	<0.001
	Age	3.5	2.7-4.7	<0.001	9.9	6.5-15.2	<0.001
	*MYCN*	3.3	2.5-4.4	<0.001	7.2	5.1-10.1	<0.001
	*SLC19A1*	2.0	1.5-2.6	<0.001	3.7	2.5-5.4	<0.001
**multivariate**	Stage	2.0	1.5-2.7	<0.001	2.8	1.8-4.3	<0.001
	Age	2.2	1.6-3.1	<0.001	4.4	2.7-7.0	<0.001
	*MYCN*	1.6	1.2-2.3	0.005	2.5	1.7-3.6	<0.001
	*SLC19A1*	1.4	1.0-1.9	0.054	1.8	1.2-2.8	0.006

Variables adjusted for in the multivariate analysis included Stage (tumor INSS Stage 1, 2, 3, 4S *vs* 4), Age (age at diagnosis ≤18 months *vs* > 18 months), *MYCN* (amplified *vs* non-amplified), and *SLC19A1* (mRNA expression dichotomized at the median).

## DISCUSSION

Altered metabolism is a defining feature of tumor cells [23, 24]. Activation of the Myc oncogene in particular reprograms metabolic pathways, providing increased biosynthetic and bioenergetic capacity [25] through upregulation of protein synthesis [26], glycolysis [27], glutaminolysis [28], nucleotide synthesis [29] and polyamine synthesis [21], all of which contribute to rapid proliferation. Genes of the folate-mediated one-carbon metabolic pathway have previously been shown to be upregulated with c-Myc expression [30] and to be correlated with neuroblastoma aggressiveness in bioinformatics analyses [13]. Furthermore, tumors with upregulated folate metabolism have been proposed to be sensitive to folate antagonists [13, 31]. The current study extends these observations to demonstrate experimentally that *MYCN*-amplified neuroblastoma cell lines have increased folate requirements and are more sensitive to methotrexate treatment than their non-amplified counterparts. Interestingly, enhanced folate requirements and methotrexate sensitivity were not simply a consequence of more rapid proliferation in *MYCN*-amplified cell lines, as the proliferation rates of *MYCN*-amplified and non-amplified neuroblastoma lines were not significantly different across the cell line panel despite the colony assays being conducted over a timeframe well in excess of cell doubling times.

While there is a clear increase in methotrexate sensitivity with *MYCN* amplification in cell lines, a key question is whether metabolic changes associated with *MYCN* amplification can be targeted clinically. Despite the widespread use of methotrexate as a chemotherapeutic agent to treat a variety of haematological malignancies and solid tumors [4], methotrexate is not routinely used to treat neuroblastoma, largely due to high rates of toxicity and low response rates observed in clinical trials conducted in the 1970s. Of these studies, one reported toxicity in three neuroblastoma patients treated with methotrexate and observed no apparent therapeutic responses [6]. The second study investigated the use of methotrexate in 16 patients with previously treated metastatic neuroblastoma and reported no objective responses, despite a majority of patients achieving plasma methotrexate concentrations exceeding 5 μM [5]. No subsequent clinical trials have examined the efficacy of methotrexate in children diagnosed with neuroblastoma. While these studies were performed prior to the prognostic use of *MYCN* amplification and therefore did not consider *MYCN* amplification status, *MYCN* amplification is now known to occur in approximately 20% of primary neuroblastomas [7, 8], and is likely to be over-represented in a trial enrolling patients with metastatic disease [32]. Knowledge of *MYCN* amplification status alone may not be sufficient to determine those patients most likely to respond to antifolate therapy, and assessment of any potential clinical application of these findings will also have to consider the limitations of methotrexate toxicity and methotrexate availability in the tumor.

To this end, quantification of *SLC19A1* expression may be of additional benefit. RFC, encoded by *SLC19A1*, is the principle cellular uptake mechanism for methotrexate [3] and *SLC19A1* mutations or altered RFC expression impact on methotrexate uptake and sensitivity [14-16, 33]. We found that down-regulation of *SLC19A1* substantially decreases the rate of methotrexate uptake in neuroblastoma cells, indicating that RFC is also a major methotrexate uptake mechanism in neuroblastoma. Furthermore, we found that *SLC19A1* expression is upregulated by N-Myc, and that high *SLC19A1* expression is correlated with neuroblastoma outcome, raising the possibility that a subset of high-risk patients with *MYCN* amplification and high *SLC19A1* expression might be appropriate candidates for methotrexate therapy.

These findings may also extend beyond *MYCN*-amplified neuroblastoma, since *MYC* family genes, including *MYCN*, *MYC* and *MYCL1* appear to be dysregulated in some high-risk neuroblastomas that lack *MYCN* amplification. Recent studies correlating microarray signals from *MYC* target genes with the clinical outcome of neuroblastoma found that *MYC* transcriptional signatures are predictive of disease outcome independent of *MYCN* amplification status [34, 35]. As *SLC19A1* has previously been identified as a *Myc* target gene [17], there may also be a subset of non-amplified tumours with highly efficient methotrexate uptake.

The findings presented in this study, although preliminary, provide new evidence to suggest that *MYCN* amplification leads to antifolate sensitivity, and that methotrexate may be beneficial in a subset of patients with *MYCN*-amplified neuroblastoma and high *SLC19A1* expression. These observations warrant further investigation in pre-clinical animal models and may also have relevance for other tumor types where *MYC or MYCN* amplification is observed, such as breast cancer, small-cell lung cancer, retinoblastoma, medulloblastoma, rhabdomyosarcoma, and astrocytoma.

## MATERIALS AND METHODS

### Patient samples

A discovery set of 42 primary neuroblastoma samples was provided by the Tumour Bank at Children's Cancer Institute Australia and has been described elsewhere [36]. Clinical data, including age, sex, INSS stage, dates of diagnosis, relapse and death from disease and *MYCN* status were obtained from medical records. All children were treated using standard protocols according to their tumor stage as previously described [36]. Gene expression and clinical data were also obtained for an independent validation cohort of 650 neuroblastoma patients as previously published [20]. A summary of clinical characteristics for each cohort is presented in Supplementary Table S1.

### Gene Set Enrichment Analysis

Gene Set Enrichment Analysis was performed on the 650 neuroblastoma patient dataset [20] with the GSEA application (Broad Institute at MIT, Cambridge, MA) [19] using the KEGG pathway gene sets (186 gene sets). Gene sets showing a false discovery rate of 0.25 or less were considered to be significantly enriched between classes.

### Cell lines and culture conditions

BE(2)-C, LAN-1, SH-EP, and SH-SY5Y were provided by Dr June Biedler, Memorial Sloan-Kettering Cancer Centre, New York, NY and NBL-S and NBL-WN by Dr Susan Cohn, University of Chicago, IL. IMR-32 was purchased from American Type Culture Collection (Manassas, VA) and SK-N-AS, SK-N-DZ, SK-N-FI, CHP-134, Kelly, NB69 from European Collection of Cell Cultures. Cell lines were cultured in Roswell Park Memorial Institute (RPMI) 1640 with 10% FCS (Life Technologies, Carlsbad, CA) and 2mM L-glutamine (CHP-134, Kelly, NB69), Dulbecco's modified Eagle's medium (DMEM) with either 20% FCS (NBL-WN), 15% FCS (NBL-S) or 10% FCS (remaining cell lines). The identity of each cell line was verified by short tandem repeat genetic profiling (CellBank Australia, Sydney, Australia).

### Colony formation assays

Cells were depleted of endogenous folate cofactors by culturing for 10 days in folate-free depletion medium consisting of folic acid free RPMI (Life Technologies) with 10% dialysed FCS (Life Technologies) and 2mM L-glutamine, supplemented with 10 μM thymidine (Sigma-Aldrich, St Louis, MO), and 100 μM adenosine (Sigma-Aldrich) as previously described [37]. For experiments, cells were trypsinized then washed in folate-free assay medium consisting of folic acid free RPMI with 10% dialysed FCS and 2mM L-glutamine. Cells were diluted in folate-free assay medium and plated in 6-well plates at the following cell numbers: CHP-134: 600 cells/well; Kelly: 400 cells/well; NB69: 400 cells/well; SHEP: 150 cells/well supplemented with folinic acid (Sigma-Aldrich) at 0, 0.5nM, 1nM, 2.5nM, 5nM and 10nM. After 9 days (CHP-134, NB69 and SH-EP) or 12 days (Kelly), colonies were stained with 0.5% crystal violet in 50% methanol. Plates were scanned, and colonies counted. Colony numbers are normalised to 10 nM folinic acid at 100%, and comparisons made to the 5 nM folinic acid values. Results are from triplicate experiments.

### Methotrexate sensitivity assays

For cell viability assays, cells were plated in 96-well plates and treated the following day with methotrexate. Cell growth was determined after 72 h of continuous exposure to 0.1nM–1mM methotrexate using a resazurin-based assay (Sigma-Aldrich), with fluorescence measured using a Benchmark Plus plate reader (Bio-Rad). Cell doubling times were determined by plating cells in 96-well plates and measuring fluorescence as above every 24 hours for 4 days.

### Gene induction and suppression experiments

A SH-EP neuroblastoma cell line harboring an inducible *MYCN* construct (Tet21N) was provided by Dr Manfred Schwab, German Cancer Research Center, Heidelberg, Germany [10] and cultured as described previously [38]. *MYCN* expression was suppressed using 2ug/mL tetracycline, with *MYCN* induction following tetracycline removal. *MYCN*-induced and control cells were harvested 72 h after tetracycline removal. Silencing of *MYCN* and *SLC19A1* was achieved using Dharmacon ON-TARGET plus SMARTpool siRNAs (Thermo Fisher Scientific, Lafayette, CO) at 10nM with a non-targeting siRNA pool as a negative control using Lipofectamine transfection reagent (Invitrogen, CA). Cells were harvested at 72 hours post-transfection to assess gene expression.

### Protein expression analysis

For analysis of RFC protein expression, whole membrane extracts were prepared using a Mem-PER Eukaryotic Membrane Protein Extraction Reagent Kit (Thermo Fisher Scientific, Lafayette, CO). Protein was quantitated by BCA assay (Pierce, Rockford, IL) and 10 μg of each sample was electrophoresed on a 4–15% SDS-PAGE gel and transferred to a nitrocellulose membrane (Biorad, Gladesville, NSW, Australia). Membranes were blocked overnight with 5% skim milk powder in Tris-buffered saline. Membranes were incubated with rabbit polyclonal anti-RFC [39] or rabbit polyclonal Na+/K+-ATPase (H-300, sc-28800; Santa Cruz Biotechnology, Dallas, TX; 1:1000), followed by HRP-conjugated secondary antibodies. Membranes were developed using Supersignal reagent (Progen Biosciences, Brisbane, QLD, Australia), and protein expression was visualised on film.

### Methotrexate uptake assay

BE(2)-C cells were transfected with control or *SLC19A1* siRNA as described above. At 24 h post-transfection, cells were replated at 3.5×10^5^ cells/well into a 6-well plate for the [^3^H]methotrexate uptake assay, with the remainder cultured to confirm RFC protein knockdown (both at 72 h post-transfection). For the [^3^H]methotrexate uptake assay, the cell monolayer was washed once with serum-free DMEM before addition of 0.8 μCi [3′, 5′, 7′-^3^H]methotrexate sodium salt (ARC, Bioscientific; specific activity = 40Ci/mmol; final methotrexate concentration = 20nM) to each well in 1mL of serum-free DMEM. Following incubation at 37°C for the specified time, medium was removed and cell monolayers were washed 3 times with ice-cold PBS. 1mL of 0.5M NaOH was added to each well, and cells were lysed by incubation at 70°C for 1 h. 1mL of 0.5M HCl was added to each well and 60uL was removed from each well and kept aside for BCA assay with the remainder transferred to scintillation vials containing 4mL of Ultima Gold™ scintillant. DPM was counted and [^3^H]methotrexate uptake calculated as fmoles of [^3^H]methotrexate/mg protein.

### RNA isolation and mRNA expression analysis

Total RNA was extracted from primary neuroblastoma tissues and cell lines using guanidinium thiocyanate (GTC) as previously described [40]. Reverse transcription was performed with MMLV reverse transcriptase according to manufacturer's protocol (Invitrogen). Quantitative PCR (QPCR) was performed using TaqMan gene expression assay (Applied Biosystems, CA). For the discovery set, expression levels of *MYCN* (primers and probe listed in Supplementary Table S2) and *SLC19A1* (inventoried TaqMan assay Hs00953342_m1) were quantified using the comparative threshold cycle (Ct) method and normalized to TaqMan endogenous control assay for β2-microglobulin (*B_2_M*; B2M Endogenous control, VIC/MGB probe, primer limited), and expressed relative to a calibrator [41]. Gene expression data from the 650 patient validation cohort was previously described [20] and was derived from single-color gene expression profiles using 44K oligonucleotide microarrays as described previously [42]. Data were log2 transformed and zero centred.

### ChIP and luciferase reporter gene assays

Putative N-Myc binding sites, including the canonical E-box sequence CACGTG and other non-canonical sequences (CATGTG and CACGCG) within the promoter region of *SLC19A1* were identified using bioinformatics techniques as previously described [43]. Binding sites located within 2000 base pairs either side of the transcription start site of the *SLC19A1* gene (as annotated in publically available databases) were chosen for further analysis, as well as a site outside of this region. To assess binding of N-Myc to *SLC19A1*, quantitative ChIP was performed from SH-EP Tet21N cells cultured with or without tetracycline, as well as from BE(2)-C cells, as previously described [43, 44]. *APEX-1* [17] and *ABCA10* [22] served as positive and negative controls. Specific primers used for ChIP are listed in Supplementary Table S2. A fragment of DNA containing each of the putative binding sites within 2000 base pairs either side of the *SLC19A1* transcription start site was cloned upstream of a luciferase reporter gene. Transcription activity was tested in SH-EP Tet21N cells grown with or without tetracycline, with *ABCC1* used as a positive control [22].

### Statistical analyses

Statistical analyses utilising patient data were performed using STATA version 10 (StataCorp, College Station, TX). Expression levels of *SLC19A1* in tumor samples were stratified into two groups around the median and designated as “high” or “low”. The frequencies of INSS stage, age at diagnosis, sex and *MYCN* status in these two groups were examined using Fisher's exact test. Correlation between *MYCN* and *SLC19A1* mRNA levels in tumor samples and cell lines was evaluated using Spearman's rank correlation coefficient. EFS was defined as the time to relapse or death within 5 years from initial diagnosis, while OS was defined as the time to death within 5 years from initial diagnosis. The cumulative EFS and OS were computed by the Kaplan-Meier method and compared between subgroups using the log-rank test. A Cox proportional hazards model was used to determine whether the expression of *SLC19A1* as well as established prognostic factors (*MYCN* amplification, neuroblastoma INSS stage, and age at diagnosis using an age cut-off of 18 months) were predictive of EFS or OS. Probabilities of survival and hazard ratios are presented with 95% confidence intervals (CIs).

All other statistical analyses were conducted using Prism version 6 (GraphPad Software, La Jolla, CA). Cell doubling times were calculated using an exponential growth equation. [^3^H]methotrexate uptake was analysed using a one-phase association equation with *P* values for determining significant differences in the rate constant (K) determined using the extra sum-of-squares F test.

Gene expression in *MYCN*-induced or silenced cells was compared using paired *t*-tests with two-sided *P* values. Dose-response curves were fitted with a three parameter logistic model by non-linear regression in order to calculate methotrexate IC_50_ and EC_50_ (half-maximal inhibition) values. *P* values for determining significant differences in IC_50_ values were determined using the extra sum-of-squares F test. Colony assay *P* values were determined using one-way ANOVA, with individual comparisons assessed using Dunnett's multiple comparison test.

## SUPPLEMENTARY MATERIALS TABLES AND FIGURES


