# Supplementary material for: MYCN amplification confers enhanced folate dependence and methotrexate sensitivity in neuroblastoma
Source: Oncotarget. 2015 Mar 30;6(17):15510–23. doi: 10.18632/oncotarget.3732 (PMC4558167; doi:10.18632/oncotarget.3732)
Supplement: Supplementary file 1 [file oncotarget-06-15510-s001.pdf]

MYCN amplification confers enhanced folate dependence and methotrexate sensitivity in neuroblastoma

Supplementary Material

Supplementary Table S1: Clinical characteristics of the 42 patient discovery cohort and the independent validation cohort of 650 patients.

	discovery cohort		validation cohort	
**Characteristics**	**Total**	**%**	**Total**	**%**
Total study participants	42		650	
Age at diagnosis				
≤ 18 months	24	57	414	64
> 18 months	18	43	236	36
Median age at diagnosis	1.32 years		1.10 years	
range	0.07–5.81 years		0.97–1.23 years	
Sex				
Male	24	57	345	56
Female	18	43	268	44
MYCN				
Single copy	30	71	550	85
Amplified	12	29	99	15
INSS stage				
Stage 1,2,3,4S	24	57	430	67
Stage 4	18	43	215	33
No. of Events[a]	19	45	218	35

[a]Events = relapse or death attributed to neuroblastoma within 5 years of initial diagnosis for both the discovery cohort and the validation cohort.

Supplementary Table S2: DNA sequences for primers used for quantitative rtPCR

**Primer**	**Primer sequence (5' to 3')**
MYCN forward	CGACCACAAGGCCCTCAGTA
MYCN reverse	CAGCCTTGGTGTTGGAGGAG
MYCN probe	6-FAM-CGCTTCTCCACAGTGACCACGTCG
SLC19A1-A F	CTAGATTACAACTGCCAAGTGCTG
SLC19A1-A R	CAGGTGTAGCCGAGCGAATC
SLC19A1-B F	GCGGATTGTGCTGTCTACAC
SLC19A1-B R	CTAAGGAGGAGCCAGGAAGTC
SLC19A1-C F	GCCAAAAGGAAAACGCACAAGG
SLC19A1-C R	TCTCCCCACCCGGTCACC
SLC19A1-D F	GGGCTGCGGGGTGTCTCG
SLC19A1-D R	CCCACACTCACCTCACAGG
APEX1 F	GCATCTGGCACAACGATAAACA
APEX1 R	GCGTGATTGGTCTGTCTGACA
ABCA10 F	AGCAACATCACCAACCTTATATTTCCC
ABCA10 R	TTAGTCAGTAAACACTCACTCAGTAAAGC

Supplementary Table S3: KEGG pathways positively correlated with *MYCN* amplification from the 650-patients Kocak cohort.

KEGG pathway name	Size	ES[a]	FDR q-val[b]	Rank at max[c]
Ribosome	86	0.752	0.041	3119
DNA_replication	34	0.749	0.022	3632
Base_excision_repair	33	0.703	0.049	3159
One_carbon_pool_by_folate	17	0.692	0.047	1030
RNA_polymerase	28	0.681	0.049	2554
Homologous_recombination	28	0.648	0.077	3632
Nucleotide_excision_repair	43	0.621	0.108	4360
Mismatch_repair	23	0.617	0.101	3768
Glyoxylate_and_dicarboxylate_metabolism	15	0.613	0.095	1035
Aminoacyl_tRNA_biosynthesis	41	0.613	0.087	2613
RNA_degradation	54	0.611	0.081	4495
Cysteine_and_methionine_metabolism	33	0.590	0.103	1190
Spliceosome	116	0.586	0.100	5066
Pyrimidine_metabolism	92	0.583	0.098	2667
Selenoamino_acid_metabolism	25	0.560	0.128	2411
Lysine_degradation	44	0.532	0.172	2655
Biosynthesis_of_unsaturated_fatty_acids	20	0.503	0.231	4089
Purine_metabolism	151	0.500	0.224	3877
Glycine_serine_and_threonine_metabolism	31	0.496	0.225	4109
Cell_cycle	121	0.489	0.231	2796

[a]ES = Enrichment Score

[b]FDR q-val = False Discovery Rate

[c]Rank at max = position in the ranked list at which the maximum enrichment score occurred.

Supplementary Table S4: Doubling times and methotrexate sensitivity measurements for *MYCN*-amplified and non-amplified neuroblastoma cell lines.

Cell line	Doubling time (d) (95%CI)	IC_{50} (μM) (95%CI)	EC_{50}[a] (μM) (95%CI)	maximal response[b] (% survival) (95%CI)
***MYCN* amplified**				
BE(2)-C	2.06 (1.57–3.00)	0.59 (0.2–1.51)	0.17 (0.06–0.52)	39.1 (32.3–45.9)
IMR-32	2.01 (1.58–2.74)	0.21 (0.12–0.4)	0.33 (0.15–0.71)	–1.92 (–10.1–6.29)
LAN-1	1.22 (1.02–1.54)	1.06 (0.44–2.51)	0.22 (0.09–0.52)	40.5 (34.6–46.4)
NBL-WN	2.83 (1.99–4.88)	1.18 (0.6–2.07)	0.39 (0.19–0.81)	30.3 (22.9–37.7)
SK-N-DZ	1.93 (1.34–3.43)	0.12 (0.1–0.15)	0.09 (0.07–0.12)	16.7 (14.4–18.9)
CHP-134	1.84 (1.49–2.42)	0.05 (0.03–0.08)	0.05 (0.03–0.09)	–3.69 (–12.3–4.89)
Kelly	2.62 (1.42–17.0)	0.06 (0.04–0.09)	0.04 (0.02–0.07)	15.4 (7.86–22.8)
***MYCN* single copy**				
NBL-S	2.80 (2.09–4.24)	>1000[c]	0.67 (0.39–1.15)	51.6 (47.4–55.8)
SH-EP	1.28 (1.04–1.65)	>1000	0.03 (0.01–0.08)	66.3 (63.0–69.6)
SH-SY5Y	2.02 (1.45–3.34)	>1000	0.42 (0.15–1.13)	64.1 (57.0–71.1)
SK-N-AS	2.46 (1.81–3.84)	>1000	0.06 (0.01–0.34)	70.3 (62.8–77.7)
SK-N-FI	3.21 (1.91–9.91)	>1000	1.86 (0.01–34.7)	83.2 (72.6–93.9)
NB69	3.50 (2.05–11.9)	>1000	0.09 (0.058–0.13)	50.6 (46.7–54.5)

[a]EC_{50} = half-maximal response (relative IC_{50})
[b]Maximal response fitted dose-response curves at 1mM methotrexate
[c]IC_{50} was not reached by the maximum methotrexate concentration (1mM)

Supplementary Figure S1

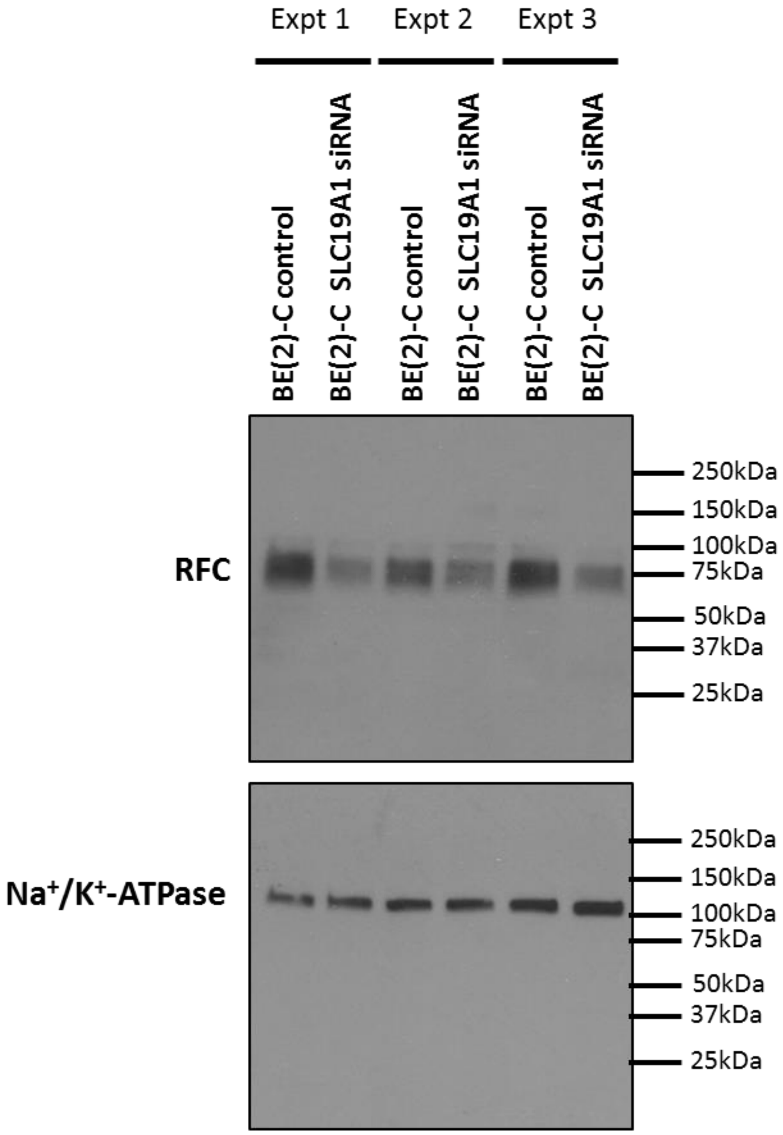

Full-length Western blots for RFC and Na^+/K^+-ATPase loading control in control and SLC19A1 siRNA treated BE(2)-C cells. A cropped, representative replicate is shown in Figure 2E.
